# The inability to self-evaluate smell performance. How the vividness of mental images outweighs awareness of olfactory performance

**DOI:** 10.3389/fpsyg.2015.00627

**Published:** 2015-05-18

**Authors:** Kathrin Kollndorfer, Ksenia Kowalczyk, Stefanie Nell, Jacqueline Krajnik, Christian A. Mueller, Veronika Schöpf

**Affiliations:** ^1^Department of Biomedical Imaging and Image-guided Therapy, Medical University of Vienna, Vienna, Austria; ^2^Department of Otorhinolaryngology, Medical University of Vienna, Vienna, Austria; ^3^Department of Psychology, University of Graz, Graz, Austria; ^4^BioTechMed, Graz, Austria

**Keywords:** olfaction, self-evaluation, olfactory dysfunction, olfactory imagery

## Abstract

To rate one’s individual olfactory performance is difficult and in many cases differs clearly from validated objective olfactory performance measures. This study aimed to investigate the basis for this measurement drift between objective and subjective olfactory performance evaluation. In absence of an actual odor, one may imagine an olfactory stimulus to evaluate his subjective olfactory performance. Therefore, the impact of the vividness of mental images on self-evaluation of smell performance in patients with mild to severe olfactory dysfunction and healthy controls was investigated. Fifty-nine patients with peripheral olfactory dysfunction ranging from reduced olfactory function (hyposmia) to complete loss of olfactory perception (anosmia) and 16 healthy controls were included. Olfactory performance was assessed using the Sniffin’ Sticks battery, the vividness of olfactory mental images was evaluated using the vividness of olfactory imagery questionnaire (VOIQ). Decreased vividness of odor images was obtained for anosmic patients, and a trend of poorer odor imagery was determined in hyposmic patients. Multiple regression analyses revealed the VOIQ score as significant predictor for olfactory self-evaluation for hyposmic patients and healthy controls. In contrast, for anosmic patients, the only significant predictor for self-rating of olfactory performance was the threshold-detection-identification (TDI) score, measuring overall olfactory performance. The results of this study indicate that sensory perception and mental images are closely related to each other. Furthermore, subjects who were able to perceive odors, even to a smaller extent, rely on the vividness of their mental odor images to evaluate their olfactory performance. In contrast, anosmic patients rather trust in their knowledge that they are not able to perceive odors. We are therefore able to subjectively rate our olfactory performance levels, if we are not able to perceive odors, but not if we are able to perceive olfactory input.

## Introduction

Previous studies on the ability of a self-assessment of overall olfactory function provided ambiguous results. It has been shown that patients with smell loss are often unaware of their olfactory deficits (Nordin et al., 1995; Murphy et al., 2002). However, some results suggest that patients with severe smell loss are more aware of their dysfunction compared to hyposmic patients (Schöpf and Kollndorfer, 2015). Also healthy controls seem to be challenged by self-ratings of their olfactory performance (Landis et al., 2003). Thus far, little is known on the origins of difficulties in the self-evaluation of olfactory function. The major question regarding the challenge of self-evaluation of olfactory abilities is the absence of current odors during self-evaluation. One possible strategy of self-rating one’s own olfactory abilities without a current odor as basis of assessment is the retrieval of mental odor representations perceived in the past.

Mental odor representations—or mental images—are defined as the creation of mental representations in absence of an external stimulus (Freeman, 1981). Mental imagery has been well documented in a broad range of sensory systems: visual (Farah, 1989; Kosslyn et al., 2001), auditory (Halpern and Zatorre, 1999), and motor system (Jeannerod and Frak, 1999). The evidence for the ability to form mental odor representations without any olfactory stimulus has been discussed controversially. Even though some researchers suppose inability to form mental odor representations (Engen, 1987; Herz, 2000), support for olfactory imagery is available from research in olfactory hallucinations (Arguedas et al., 2012), dreams (Stevenson and Case, 2005a), and volitional imagery (Djordjevic et al., 2004). Mental imagery is often assessed by vividness ratings. In these questionnaires subjects are instructed to create mental representations for a certain sensory system and to evaluate the degree to which the mental representation equals the perceptual experience (Sheehan, 1993; Gilbert and Kemp, 1996). However, it has already been reported that the imagination of odors occurs less frequently and is less vivid than the imagination of other senses, e.g., sights or sounds (for review, see Stevenson and Case, 2005b; Arshamian and Larsson, 2014). Thus far, less is known about the vividness of mental odor representations in patients with olfactory dysfunction.

Therefore, the present study aimed to investigate the impact of the vividness of mental odor representations on the ability to evaluate one’s own olfactory performance. Therefore, we investigated patients with mild to severe olfactory dysfunction and healthy controls. We hypothesized that the ability to generate vivid olfactory representations is reduced in patients with olfactory dysfunction, as supposed by the perceptual theory. Furthermore, we assume that the vividness of mental representations influences self-ratings of olfactory performance.

## Materials and Methods

### Subjects

Ninety-two patients with olfactory dysfunction were initially included in this study. To avoid interference with memory, or other cognitive impairment in patients with traumatic brain injury, only patients with smell loss due to sinonasal diseases or idiopathic olfactory dysfunction were included in our final sample. Our cohort therefore consisted of 59 patients with olfactory dysfunction (34 female, 25 male) and 16 healthy controls (nine female, seven male). Information on this control groups olfactory performance has already been presented in Krajnik et al. (2014). Detailed sociodemographic data, is presented in Table 1. The study was designed as a retrospective data analysis study investigating vividness of olfactory imagery on a selected group (olfactory dysfunction) of a large study population that was acquired in a different context, but with the questionnaires necessary for the present paper. All subjects had no history of neurologic or psychiatric diseases. The study was approved by the Ethics Committee of the Medical University of Vienna. All subjects were informed about the aim of the study and gave their written informed consent prior to inclusion.

**TABLE 1:** **Sociodemographic data of the study sample**.

	**Olfactory dysfunction**		**Healthy controls**
	**Anosmics**	**Hyposmics**	
	**Mean (SD)**	**Mean (SD)**	**Mean (SD)**
Number of participants (male/female)	43 (19/24)	16 (6/10)	16 (7/9)
Age	54.09 (13.60)	56.13 (8.62)	30.63 (6.98)
Duration of smell disorder (in years)	9.43 (10.05)	12.97 (14.07)	–

### Olfactory Performance

Olfactory performance was assessed using the Sniffin’ Sticks test battery (Burghart Instruments, Wedel, Germany). This test battery includes three subtests that assess nasal chemosensory function: detection threshold; odor discrimination; and odor identification. The Sniffin’ Sticks battery uses pen-like devices for odor presentation (Kobal et al., 1996, 2000; Hummel et al., 1997). The odor detection threshold of *n*-butanol was identified using a single-staircase, three-alternative, forced-choice procedure. In the second subtest, odor discrimination ability was determined using 16 triplets of odorants (two pens contained the same odorant; the third pen contained an odd odorant). The participants were asked to detect the odd pen in a forced-choice procedure. The odor identification task consists of 16 common odors using a multiple-choice answering format, with a list of four descriptors for each odor, again in a forced-choice procedure. The scores for the detection threshold range from 1 to 16, and, for the other two subtests, a score between 0 and 16 may be achieved. The results of all three subtests were summed to obtain the threshold-detection-identification (TDI) score. Normosmia, or normal olfactory performance, is characterized by a TDI score of at least 31, and hyposmia (reduced olfactory function) is defined as a TDI between 17 and 30.75. A TDI-score of less than 17 is categorized as anosmia (Kobal et al., 2000). In addition, all participants were asked to evaluate the 16 odors of the identification test regarding their intensity of the odor (1 = very weak; 9 = very intense). Furthermore, all subjects were asked to evaluate their sense of smell on a nine-point scale (1 = good sense of smell; 9 = poor sense of smell).

### Olfactory Imagery

The capability for olfactory imagery was assessed with the *vividness of olfactory imagery questionnaire* (VOIQ; Gilbert et al., 1998), translated into German (see supplementary materials). The participants were instructed to mentally retrieve 16 odors of four different categories: personal hygiene (bath); food-related (barbecue); tobacco; and vehicles (car). In each category, the subjects were verbally presented with four specific odors and were asked to imagine (e.g., “The odor of unlit tobacco—a cigarette, cigar, or pouch of pipe tobacco.”). For each specific situation, the participants had to evaluate the vividness of their imagination on a five-point Likert scale (1 = perfectly realistic and as vivid as the real odor; 5 = No odor at all, you only “know” that you are thinking of an odor). All 16 items were summed to a total score, with low values reflecting good odor imagery abilities, and high values representing poor olfactory imagination abilities. In addition, total values were calculated for each category.

### Data Analysis

Statistical analysis was performed using the Statistical Package for the Social Sciences (SPSS, Chicago, Illinois), version 20.0. For all test scores, mean and standard deviation (SD) were calculated. To investigate the impact of olfactory impairment on odor imagery, anosmic and hyposmic patients were compared with healthy controls. All variables fulfilled requirements for parametric testing, thus Pearson’s correlation, and one-way ANOVA were calculated. *Post hoc* Tests were Bonferroni-corrected to deal with alpha-inflation. Equality of variances was calculated using the Levene-Test. Group differences in self-evaluation of olfactory performance were calculated using the non-parametric Kruskal–Wallis test. Correlations between self-evaluation and TDI scores were computed using Spearman’s rho. For all reported variables, variances did not differ significantly. Multiple regression analyses were computed to figure out potential predictors for self-evaluation of olfactory performance for all three groups separately. The alpha level for all statistical tests was set to α = 0.05.

## Results

### Sociodemographic Data

The sample was tested for significant differences in gender distribution and educational background. For all three groups, anosmics, hyposmic, and normosmics no differences for gender (χ^2^ = 0.223; *p* = 0.895) and educational background (χ^2^ = 6.541; *p* = 0.365) were determined. With regard to age, significant group differences were determined between healthy controls and patients with olfactory dysfunction [*F*(2,72) = 27.374; *p* < 0.001]. *Post hoc* analysis revealed no difference in age between anosmic and hyposmic patients (*p* = 0.999).

### Olfactory Performance

Data analysis revealed a mean TDI score for anosmic patients of 11.97 (SD 2.74). Participants with reduced olfactory function achieved a mean TDI score of 24.25 (SD 3.71). For the healthy control group, a mean TDI score of 35.80 (SD 2.23) was obtained. Mean TDI values of the three groups differed significantly [*F*(2,72) = 423.48; *p* < 0.001; ${\text{η}}_{p}^{2}$ = 0.922]. Detailed olfactory performance results and subjective evaluation of olfactory performance are summarized in Table 2.

**TABLE 2:** **Results of olfactory performance measures**.

	**Olfactory dysfunction**		**Healthy controls**	
	**Anosmics Mean (SD)**	**Hyposmics Mean (SD)**	**Mean (SD)**	***p*-value**
Odor threshold	1.45 (0.89)	4.44 (2.69)	9.05 (1.78)	<0.001
Odor discrimination	5.567 (1.91)	9.68 (1.58)	12.94 (1.69)	<0.001
Odor identification	4.97 (1.96)	10.13 (3.36)	13.81 (1.42)	<0.001
TDI score	12.97 (2.74)	24.25 (3.71)	35.80 (2.23)	<0.001
Subjective olfactory performance	8.51 (0.77)	6.93 (1.69)	3.06 (1.79)	<0.001
Intensity rating	1.72 (1.63)	4.05 (1.61)	7.22 (1.14)	<0.001

### Olfactory Imagery

**FIGURE 1:**
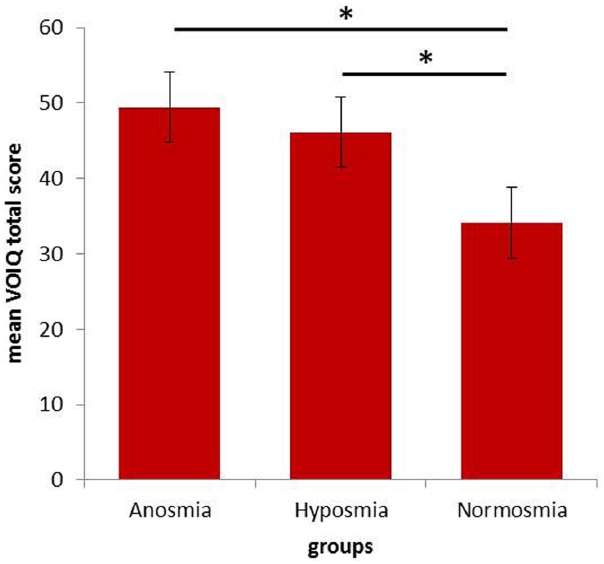
**Mean total VOIQ scores for all three groups [anosmic patients (n = 43), hyposmic patients (n = 16), and healthy controls (n = 16)].** Significant group differences (*p* < 0.05) are marked with an asterisk. Error bars reflect the standard error.

Analysis of the VOIQ total score revealed significant group differences [*F*(2,72) = 6.667; *p* = 0.002; ${\text{η}}_{p}^{2}$ = 0.156]. *Post hoc* analyses showed significantly higher VOIQ scores in patients with olfactory dysfunction compared to healthy controls (see Figure 1). A detailed overview of olfactory imagery performance is presented in Table 3. Hyposmic patients did not differ significantly from the two other subject groups in their VOIQ score. However, a trend of poorer vividness of mental representations in hyposmic patients compared to healthy controls (*p* = 0.065) was observed. Even though the healthy control group was significantly younger compared to anosmic and hyposmic patients, neither age (*r* = 0.149, *p* = 0.203) nor gender (*r* = 0.067, *p* = 0.566) influenced the VOIQ performance significantly. Investigating the influence of duration of olfactory dysfunction, no significant correlation was determined for patients with olfactory dysfunction (*r* = 0.115, *p* = 0.387).

**TABLE 3:** **Results of olfactory imagery questionnaire for vividness (VOIQ)**.

	**Olfactory dysfunction**		**Healthy controls**	
	**Anosmics Mean (SD)**	**Hyposmics Mean (SD)**	**Mean (SD)**	***p*-value**
VOIQ—bath	12.60 (4.56)	11.75 (3.62)	8.13 (3.16)	0.002
VOIQ—barbecue	12.72 (4.28)	12.00 (3.81)	10.13 (3.60)	0.098
VOIQ—tobacco	11.26 (4.83)	10.94 (4.37)	7.38 (3.14)	0.006
VOIQ—car	12.88 (3.89)	11.44 (3.54)	8.81 (3.60)	0.013
VOIQ—total	49.46 (15.04)	46.13 (13.24)	34.44 (11.92)	0.002

### Self-Evaluation of Olfactory Performance

The three groups differed significantly in their self-evaluation (*H* = 47.002; *p* < 0.001). *Post hoc* analyses showed significant differences between all three groups. Anosmic patients reported poorest olfactory abilities (mean 8.51; SD 0.77), whereas healthy controls reported highest olfactory abilities (mean 3.06; SD 1.79). Self-reporting data revealed that healthy controls rated themselves significantly better than hyposmics, and hyposmics rated themselves significantly better than anosmic patients. However, self-reporting data was not correlated with objective olfactory performance measurement (TDI score) in healthy controls (ρ = –0.221, *p* = 0.411) and hyposmic patients (ρ = 0.126, *p* = 0.643). Only for anosmic patients, a significant correlation between self-reporting and olfactory performance measures was obtained (ρ = –0.373; *p* = 0.014).

### Multiple Regression

**FIGURE 2:**
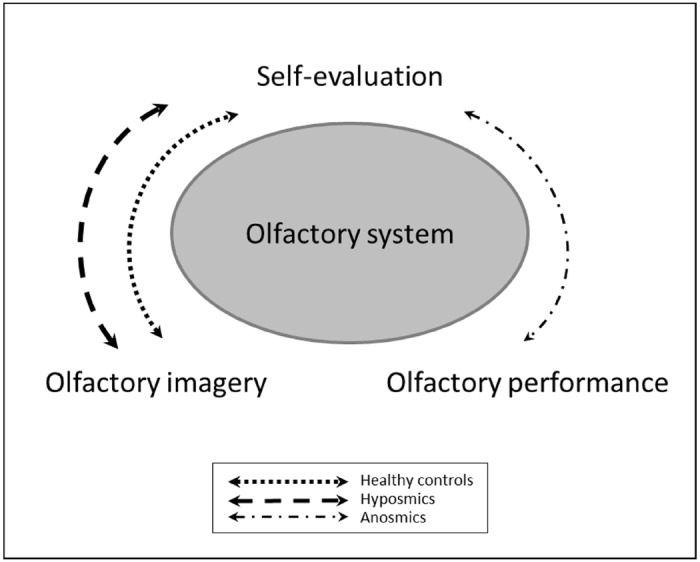
**Schematic representation of the multiple regression models for the three subject groups.** Healthy controls and hyposmic patients seem to rely on their ability to create mental representation of odors to self-evaluate their olfactory performance in absence of a current odor. In contrast, anosmic patients, rather trust in their knowledge that they are not able to perceive odors.

The multiple regression model was set up to investigate the predictors of olfactory imagery performance in more detail. In a first step, potential predictors of the dependent variable were included into the model. Following potential predictors were included in the model using stepwise iterations in multiple regression analyses: VOIQ total, TDI, score, gender, and age. Interestingly, computed statistical models differed between patients with complete smell loss and subjects who were still able to perceive odors (hyposmic patients and healthy controls). For anosmic patients, the results of the regression revealed the TDI as the only statistically significant predictor for self-evaluation of olfactory performance [*R*^2^ = 0.10; *F*(1,41) = 4.274; β = –0.307; *p* = 0.045; see Figure 2]. For the other two subject groups, not the TDI but the VOIQ score was determined to significantly predict olfactory self-rating [hyposmics: *R*^2^ = 0.33; *F*(1,14) = 6.905; β = 0.575; *p* = 0.020; healthy controls: *R*^2^ = 0.29; *F*(1,14) = 5.738; β = 0.539; *p* = 0.031; see Figure 2].

## Discussion

The present study aimed to investigate the impact of vividness of olfactory imagery on self-assessment of olfactory performance in patients with peripheral impaired olfactory function, compared to healthy controls. Results revealed significantly reduced olfactory imagery abilities in anosmic patients and a trend of poorer vividness of mental representations in the hyposmic subject group. Furthermore, different predictors for self-evaluation were obtained. In hyposmic patients and healthy controls the VOIQ score was determined as a significant predictor for olfactory self-rating. In contrast, in the anosmic patient group, the TDI score, measuring overall olfactory performance, was the only variable, that significantly predicted olfactory self-rating.

Decreased mental imagery abilities in patients with sensory loss have already been determined in visually impaired patients. Various case studies investigating cortically blind patients found impaired visual mental imagery (Farah et al., 1988; Chatterjee and Southwood, 1995; Policardi et al., 1996). These deficits in building mental images have not only been found in patients with complete sensory loss, but also in patients with impaired sensory perception. In patients with peripheral visual impairment who were still able to perceive stimuli Palermo et al. (2013) investigated the vividness of visual mental images and revealed that the presence of a visual defect, even if correted by lenses, corresponded to a decrease in the vividness of mental images. These findings are in line with our study, in which anosmic patients revealed statistically significant poorer vividness of mental representations and the hyposmic group showed a trend of reduced vividness of olfactory representations compared to healthy controls. No correlation between disease duration and vividness of olfactory imagery was obtained in patients with smell loss. We assume that the ability to imagine odors is disturbed, in patients with a decreased olfactory sensory input. Even though it is assumed that odor representations are stored predominantly in long-term memory (Herz and Engen, 1996), a continuous sensory stimulation may be required to sustain the trace of the representation. Previous studies indicate that olfactory memory is not based on internal mental representations of odors (Köster et al., 2014a). Moreover, the authors assume that olfaction is hardly comparable to other senses, such as vision due to the different functions of these senses for human beings. Whereas vision provides information on spatial orientation, olfaction is directed at warning as well as the detection of unknown and potential dangers. Therefore, the visual model of memory and recognition may not be appropriate to describe olfactory memory. This assumption is supported by a study investigating food memory. In contrast to the traditional view on visual memory as a reactivation of previous experiences, food memory is rather targeted at detecting novelty and change (Morin-Audebrand et al., 2012).

In a study which investigated how olfactory imagery is represented neurally in patients with acquired olfactory loss, a decrease in the vividness of olfactory imagery in patients with olfactory dysfunction was detected (Flohr et al., 2014). As no differences in the ability to create visual mental images were determined, compared to healthy controls, the authors concluded that regular exposure to sensory-specific stimuli is necessary to maintain the capability for mental imagery. The study sample investigated by Flohr et al. (2014) included patients with various causes of olfactory loss, with the majority of causes being traumatic brain injuries. As traumatic brain injury could not only impair the perception of odors, but also the olfactory memory, our study included only patients with peripheral olfactory dysfunction, to investigate a study sample as homogeneous as possible.

Mental odor imagery has been discussed controversially in the past. Previous studies claimed that the creation of mental representations of odors by non-experts is not possible at all (e.g., Herz, 2000). In contrast, recent investigations using functional imaging methods indicate the ability to imagine odors not only in experts (Plailly et al., 2012) but even in non-experts, as they observed an activation of the piriform cortex, the major primary olfactory area (Djordjevic et al., 2005; Bensafi et al., 2007). However, Royet et al. (2013) noted that the activation of the piriform cortex may be caused by other reasons than olfactory imagery: First, the activation of the piriform cortex may arise due to sniffing activities during an olfactory imagery task. Second, activation of the piriform cortex may be caused by drawing attention to odors in the environment of the subject. And third, the activation of the piriform cortex may be a result of cross-modal associative learning (Gottfried et al., 2002).

The sensory system is a closed mechanism, in which different variables and factors interact with each other. Rather than investigating the effect of a single parameter in an isolated way, there is the need to explore the whole system to seek understanding of its mechanisms. We therefore used multiple regression analyses in which we included measures that may influence self-evaluation. This systematic investigation revealed different predictors for self-evaluation of olfactory performance for anosmic patients compared to hyposmic patients and healthy controls. Anosmic patients, who suffer from a complete loss of their sense of smell, use the information that they perceived no odors to evaluate their own olfactory performance. In contrast, participants who are still able to perceive odors, even in smaller dimension, rather rely on their ability to imagine odors to assess their own olfactory performance. We can therefore hypothesize that if a person, who is able to perceive odors, is asked to self-evaluate their olfactory function, they will try to assess a concrete stimulus. If no odor is actually available, they might rely on the vividness of odors retrieved from long-term memory. Patients with a complete smell loss are usually aware of their inability to perceive odors and therefore trust in their knowledge of poor olfactory performance.

A potential limitation of this study is the subjective assessment of the vividness of olfactory imagery. In this study patients with olfactory dysfunction were included; therefore a comparison with actually presented odors was not possible. Previous studies claimed to assess olfactory imagery objectively (Djordjevic et al., 2004). However, imagery is always subjective, as it is a person’s rating of vividness or comparability to presented odors. Previous research (Bensafi and Rouby, 2007) has argued that the self-reporting questionnaire used in this study, is a valid measure of olfactory mental images. Furthermore, no visual imagery test was included to determine whether the difficulties in patients with olfactory dysfunction were sensory-specific or general problems with mental imagery. Based on previously published literature (for review, see Arshamian and Larsson, 2014), it can be assumed that the reduced vividness of olfactory imagery in patients with olfactory dysfunction is sensory-specific.

Another limiting factor of the present work is the healthy control group, which is significantly younger compared to anosmic and hyposmic patients. However, age and gender were neither significantly correlated with olfactory imagery nor with self-evaluation. We therefore assume that these differences do not influence the results of the present study.

## Conclusion and Future Directions

The results of our study demonstrate that the retrieval of olfactory mental representation is affected by individual olfactory performance. This study revealed that patients with peripheral smell loss show a decreased vividness of olfactory representations. Furthermore, we were able to define different predictors for olfactory self-ratings in anosmic patients compared to hyposmic patients and healthy controls. Whereas the first seem to rely rather on the fact that they do not perceive any odor to assess their own olfactory performance, the latter two subject groups tend to rely on their odor imagery abilities to evaluate their smell performance. Previous studies have already shown that olfactory training may induce significant improvements in olfactory performance (Hummel et al., 2009; Damm et al., 2014). Future studies could investigate the alterations in olfactory imagery as well as their basis of olfactory self-ratings in anosmic patients after completing olfactory training, to determine whether alterations of olfactory performance induced by such a training program are accompanied by changes in the ability to imagine odors. Although it is assumed that only about one third of general population is able to create mental odor representations, and this ability does neither improve odor identification nor odor naming abilities (Köster et al., 2014b), it can therefore be speculated, that it is unlikely that an olfactory training may force olfactory imagery abilities. However, this may be part of future investigations.

### Conflict of Interest Statement

The authors declare that the research was conducted in the absence of any commercial or financial relationships that could be construed as a potential conflict of interest.
